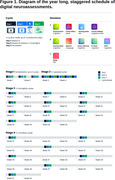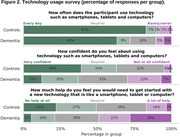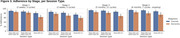# A longitudinal real‐world study in patients with Alzheimer’s Disease dementia using frequent multi‐domain digital measurements at‐home performed on the Cumulus NeuLogiq^TM^ Platform: usability and feasibility findings

**DOI:** 10.1002/alz.092164

**Published:** 2025-01-09

**Authors:** Shannon Diggin, Azar Alexander‐Sefre, Brian Murphy, Hugh Nolan, James B Rowe, Laura M Rueda‐Delgado, Alison R Buick

**Affiliations:** ^1^ Cumulus Neuroscience, Belfast UK; ^2^ Cumulus Neuroscience, Dublin Ireland; ^3^ Department of Clinical Neurosciences, University of Cambridge, Cambridge UK

## Abstract

**Background:**

Many outcome measures used in AD clinical trials require clinic visits and are paper based, making them infrequent and burdensome ‘snapshots’, subject to rater bias. A consortium of 10 pharma companies came together with Cumulus Neuroscience to design a solution for frequent, objective, real‐world measurement across a range of domains. We present a study that examined the feasibility of asking patients with mild dementia to use the neuroassessment platform repeatedly at home for one year.

**Method:**

Seven UK sites recruited Alzheimer’s type mild dementia patients (n=59, ACE‐III scores >60 and ≤88) and a matched cohort of controls (n=60). Participants had 2 weeks of at‐home familiarisation to become comfortable with the 8 assessments presented on a mobile tablet and EEG headsets, one for wake and another for sleep. A staggered longitudinal protocol followed, with burst sampling tapering to periodic sampling over the year. Benchmark paper‐based assessments (including ADAS‐Cog) and self‐reported usability were collected at months 0, 6 and 12. Additionally, plasma was collected at months 6 and 12 for later biomarker analysis.

**Result:**

Feedback collected at baseline showed differences in technology usage. Patients felt they required more technology support than controls (49% vs 17%). Nonetheless, they were successful in using the study technology at home on a regular basis and maintained a high level of adherence to the protocol. Overall compliance was 76.3% for dementia, 88.4% for controls and was highest in the latter stages of the study during periodic sampling (Stage 4 during wake EEG sessions: 80.8% dementia, 93.9% controls). System Usability Scale results indicated controls rated usability higher (63.8) than dementia participants (54.5) at baseline, increasing for both groups by Week 52 (74.4 controls; 58.3 dementia).

**Conclusion:**

With appropriate technology design, and provision of training and support, patients with Alzheimer’s disease dementia are capable and willing to provide repeated, real‐world samples of a broad range of objective digital endpoints for clinical research.